# Familial aggregation of human susceptibility to co- and multiple helminth infections in a population from the Poyang Lake region, China

**DOI:** 10.1016/j.ijpara.2007.02.008

**Published:** 2007-08

**Authors:** Magda K. Ellis, Giovanna Raso, Yue-Sheng Li, Zhu Rong, Hong-Gen Chen, Donald P. McManus

**Affiliations:** a Molecular Parasitology Laboratory, Australian Centre for International and Tropical Health and Nutrition, The Queensland Institute of Medical Research and The University of Queensland, 300 Herston Road, Herston, Brisbane, Qld 4029, Australia; b Hunan Institute of Parasitic Diseases, WHO Collaborating Centre for Research and Control on Schistosomiasis in Lake Region, Yueyang, Hunan Province, PR China; c National Institute of Parasitic Diseases, Chinese Centre for Disease Control and Prevention, 207 Rui-Jin Road, Shanghai 200025, PR China; d Jiangxi Provincial Institute of Parasitic Diseases, 330046 Nanchang, PR China

**Keywords:** Variance components analysis, *Schistosoma japonicum*, *Ascaris lumbricoides*, *Trichuris trichiura*, Poly-helminth infections

## Abstract

Human helminthiases are common in China, especially in rural areas where sanitation conditions are poor. Co- and multiple infections with helminths are strikingly frequent. A cross-sectional parasitological and questionnaire survey was carried out in a population of 3205 individuals belonging to 498 families from five villages in the Poyang Lake region, Jiangxi Province, China, to assess their helminth infection status and to collect information on risk factors for infection. The prevalences for *Ascaris lumbricoides*, *Schistosoma japonicum* and *Trichuris trichiura* were 30.9%, 15.7% and 47%, respectively. Hookworm infection prevalence was low (0.7%). A significant association was observed between *A. lumbricoides* and *T. trichiura* infection, and also between *S. japonicum* and *T. trichiura* infection. Variance components analysis was undertaken to investigate the aggregation of *S. japonicum* and the soil-transmitted helminths, *A. lumbricoides* and *T. trichiura*. While *A. lumbricoides* was found to aggregate only at a household level, *T. trichiura* was shown to cluster predominantly in families. Both genetic and household effects were found to be important in determining the risk of infection with *S. japonicum*. Variance components analysis for *A. lumbricoides/T. trichiura* co-infections indicated a significant domestic environmental effect, attributable for 32.7% of the co-infection risk. Aggregation of *S. japonicum/T. trichiura* co-infection was also observed at a household level. The risk of infection with multiple helminth species, although mainly environmentally influenced, was also shown to have significant involvement of genetic and household components. The results of this study indicate that a shared household is a major contributing risk factor for helminth co-infections and emphasises the need for increased standards of sanitation and hygiene to prevent parasite transmission. Further, the results suggest that susceptibility to one helminth infection is not completely independent of another, and that there exist common genetic factors underlying infection with multiple helminth species.

## 1 Introduction

Schistosomiasis and soil transmitted helminths (STHs) are estimated to account for more than 40% of the total tropical parasite burden, excluding malaria (WHO, 1999). Indeed, STHs (*Ascaris lumbricoides*, hookworm (*Ancylostoma*, *Necator*) and *Trichuris trichiura*) are among the most common cosmopolitan geo-helminths, and are widely distributed worldwide, including China (Hotez et al., 1997). Although there is no systematic control of STHs currently operating in China, a recent national survey of the important human parasitic diseases (Anonymous, 2005) indicated a reduction in prevalence of STHs of 61%, 71% and 74% for *A. lumbricoides*, hookworm and *T. trichiura*, respectively, since a previous national survey in 1990. *Schistosoma japonicum* is highly endemic to southern China. Due to the significant level of morbidity observed in chronically infected individuals, schistosomiasis control has been a major public health focus of Chinese authorities for more than five decades. Despite initial successes, which saw numbers of infected individuals decrease by over 90%, there are still an estimated 880,000 people afflicted by schistosomiasis in endemic foci (Li and Cai, 2004). Given the high prevalence of STHs and *S. japonicum*, it is inevitable that concurrent infections occur. Indeed, several studies across Southeast Asia, South America and Sub-Saharan Africa, have described populations having significant helminth infection prevalence, with individuals harbouring multiple helminth infections being the norm (Booth et al., 1996; Guignard et al., 2000; Lili et al., 2000; Keiser et al., 2002; Waikagul et al., 2002; Tchuem Tchuenté et al., 2003; Raso et al., 2004a,b; Zhou et al., 2005).

A characteristic feature of helminth infections is an over-dispersed distribution of observed faecal egg counts whereby a minority of individuals account for the majority of the parasite burden (Bradley and May, 1978; Anderson and May, 1985). Further, individuals who experience high intensity of infection have been shown to aggregate in families. Significant clustering at both a household and familial level was demonstrated in a population endemic for *A. lumbricoides* in Nepal (Williams-Blangero et al., 1999); the heritable component in this study accounted for ∼40% of the variation observed in faecal egg counts. Predisposition to *T. trichiura* infection has also been shown to aggregate in families (Chan et al., 1994) and quantitative methods estimated a strong heritable component (25.4%) underlying susceptibility to *T. trichiura* infection intensity (Williams-Blangero et al., 2002a). Genetic susceptibility to infection and infection intensity has been observed in populations living in areas endemic for *Schistosoma mansoni* (Bethony et al., 2001, 2002a) and *S. japonicum* (Ellis et al., 2006). Although these genetic studies investigated the clustering of single helminth infections at a familial level, nothing is known of the genetics underlying multiple helminth infections. Here we describe features of a Chinese population residing in an area co-endemic for STHs and *S. japonicum* previously studied by Ellis et al. (2006). We provide insight into the factors contributing to the susceptibility to single helminth infections in this population, using a variance components approach. In addition, we believe we provide the first evidence of possible genetic effects underlying the susceptibility to co- and multiple helminth infections in humans.

## 2 Materials and methods

### 2.1 Study population

The study population comprised five administrative villages from the Poyang Lake region in Jiangxi Province, China (Ellis et al., 2006). The five villages are located at different positions on Poyang Lake; geo-coordinates of each village are as follows: Dingshan (E115.97 N29.12), Xindong (E116.69 N28.85), Fuqian (E116.42 N28.89), Aiguo (E116.37 N28.73) and Yu Feng (E116.13 N28.51). The total population consisted of 3682 individuals. Each administrative village was comprised of four to eight smaller ‘natural villages’. Each individual was allocated a personal identification code (PID) comprised of their administrative village code, natural village code, household and house member code. Children under the age of 5 years were not included.

### 2.2 Questionnaires for the collection of demographic, treatment history and pedigree data

All individuals in the study were interviewed using an existing, validated questionnaire (Ross et al., 1997). Demographic information for age, sex, education and occupation was collected as well as history of schistosomiasis, including past infection and treatment data, and water contact. Water contact was assessed by mode and frequency of contact for spring, summer and autumn (Ross et al., 1998b).

A further questionnaire was developed and used to collect pedigree data. All individuals in the study were interviewed to identify all immediate biological relatives, i.e. parents, siblings and children. Family member PIDs were recorded as well as relationship, name, sex and date of birth of the relative to ensure correct identification. Extended families were identified using the questionnaires and family trees were created for each. Five hundred and thirty-one individuals were absent from the study (deceased or non-permanent residents) but were still included as dummy relatives in order to link families further. In-laws, who were not biologically related to anyone else in a family (i.e. had no children), were not included, and thus were not used in the household analysis. When inconsistencies were identified between the questionnaires of relatives (i.e. a wrong PID code of a relative), the information was checked, where possible, or omitted from the final data analysis. The final data comprised a total of 3205 individuals, belonging to a total of 498 families which ranged in size from three to 188 and spanned as many as four generations.

### 2.3 Parasitological examination

All individuals from the study population were asked to provide two stool samples for parasitological examination using the Kato–Katz thick smear technique (Katz et al., 1972). The second stool was collected at least 72 h after the first. In order to maximise sensitivity of the technique (Ross et al., 1998a; Berhe et al., 2004), three slides were prepared from each stool sample (Ross et al., 1998a; Li et al., 1997, 2003). Each slide was read blind by experienced microscopists for the presence of *S. japonicum* and STHs (*A. lumbricoides*, hookworm and *T. trichiura*) eggs and 10% of slides were re-read for quality control by a senior microscopist (Utzinger et al., 2000; Raso et al., 2004a,b). Positive infection was defined for each parasite species if there was an egg count of one or more on any slide. This study was primarily undertaken to investigate the genetics underlying susceptibility to *S. japonicum* infection in this population (Ellis et al., 2006); as a result, intensity of infection (eggs per gram of faeces; EPG) was recorded only for *S. japonicum* and not for the STH infections. Infection intensity was calculated for *S. japonicum* as EPG. Individuals with *S. japonicum* infection were divided into five categories depending on their infection intensity: low infection (1–4 EPG); minor infection (5–12 EPG); moderate infection (13–60 EPG); high infection (61–132 EPG) and highest infection (133+ EPG).

### 2.4 Data management and statistical analysis

All collected parasitological and questionnaire data were double-entered into an Access database and cross-checked. Investigation of prevalence estimates, parasite distributions and associations was carried out in SPSS 13.0. SOLAR, a Unix-based software program, which was also used to carry out the variance components analysis (Duggirala et al., 1997; Almasy and Blangero, 1998), the models for which were described previously (Ellis et al., 2006). Family data was coded and indexed according to the file format requirements of the SOLAR software package. Both a natural log transformation (ln(EPG + 1)) and a blom transformation were applied to normalise the distribution of EPG. However, skewness and kurtosis remained significantly different from zero (*P* < 0.001). Variance components analysis is based on a regression model whereby the phenotype is assumed to be normally distributed. If this assumption is violated, the standard errors of the parameters are underestimated and can result in type I errors, thus leading to incorrect conclusions on the model of best fit. As a result, only the binary phenotype (infected versus uninfected) and not EPG was used for *S. japonicum*. Aggregation of multiple helminth infections was investigated using the number of infections with any parasite as the phenotype. For this purpose a variable was generated, with values ranging from 0 (having no infection) to 3 (harbouring three parasite species), and was treated as a continuous variable with a normal distribution (skewness = 0.477; kurtosis = −0.8). If the phenotype under investigation is dichotomous, and therefore cannot be normally distributed, a multifactorial threshold model is used that assumes: (i) that there are several factors that are involved in the disorder in question; (ii) that the effect of each individual factor is small but that the effects of each factor are additive; and (iii) once the additive effect of these factors pass a critical threshold, an individual becomes affected. This implies that the underlying liability of affection is normally distributed but once the threshold is reached, one becomes affected and the phenotype is dichotomous.

Briefly, in a variance components analysis, the total residual variance can be expressed as the sum of the additive genetic $\left(\sigma_{\text{a}}^{2}\right)$, common environment $\left(\sigma_{\text{c}}^{2}\right)$ and individual-specific environment $\left(\sigma_{\text{e}}^{2}\right)$ variances.

$$V_{\text{tot}}=\sigma_{\text{a}}^{2}+\sigma_{\text{c}}^{2}+\sigma_{\text{e}}^{2}$$

Environmental, polygenic (additive genetic effects) and common environment (in this case household) effects can thus be estimated according to the parameter constraints specified in the model. The general household/polygenic model estimates all parameters, whereas the household and the polygenic models are specified by fixing $\sigma_{\text{a}}^{2}$ and $\sigma_{\text{c}}^{2}$ at zero, respectively. Heritability can be estimated as simply the standardised value of the additive genetic effect $\left(\sigma_{\text{a}}^{2}\right)$. An advantage of using SOLAR is its ability to incorporate covariate effects into the models to account for potential confounding factors and a Kullback–Leibler R-squared value is estimated to indicate the effect size of the covariates in the model.

Here, all significant covariates (*P* < 0.05) identified in the bivariate logistic regression were included in the models as well as administration village (village effects) to account for any potential geographical differences between the study sites. The software implements a maximum likelihood method which provides a log-likelihood estimate for each model. As all models are nested on the household/polygenic model, they can then be compared with one another using a likelihood ratio test (LRT) which calculates a chi square value to be twice the difference between the likelihoods with one degree of freedom. The significance of the household and genetic effects can thus be tested individually (versus the sporadic model) and in the presence of each other (versus household/polygenic model). The model of best fit was selected as the model with most significant effects (<0.05).

### 2.5 Ethical considerations

Ethical approval for the study was granted by the ethics committees of Jiangxi Provincial Institute of Parasitic Diseases, Chinese Center for Disease Control and Prevention; National Institute of Parasitic Diseases, Chinese Center for Disease Control and Prevention, Shanghai; Hunan Institute of Parasitic Diseases, Chinese Center for Disease Control and Prevention; and the Queensland Institute of Medical Research, prior to commencement. Oral informed consent was obtained from all adults and from parents or guardians of minors (over the age of 4 years) who were involved in the project. Study participants identified as stool egg-positive for schistosomiasis were treated with 40 mg/kg of praziquantel, the current dosage recommended by the WHO. Subjects infected with STHs were advised to seek treatment from a local doctor or hospital.

## 3 Results

### 3.1 Parasite infections

Among the five administrative villages, the observed prevalences for *A*. *lumbricoides* and *T. trichiura* ranged from 0% to 38.8% (mean 30.9%), and from 2.4% to 58.1% (mean 47%), respectively, whereas the *S. japonicum* prevalence ranged from 12.2% to 20.9% (mean 15.7%). Hookworm infection prevalence was low (0.7%), and was not considered in further analysis. The difference in helminth prevalence between the administrative villages was highly significant (*χ*^2^; *P* < 0.001). Of the total population, 61.7% harboured at least one helminth species and 27.8% were identified as harbouring at least two parasite species. Again, differences between the mean number of helminth species harboured by individuals among villages were highly significant (Kruskal–Wallis test; *P* < 0.001) although the overall distribution of the number of parasite species among individuals was normal.

**Fig. 1:**
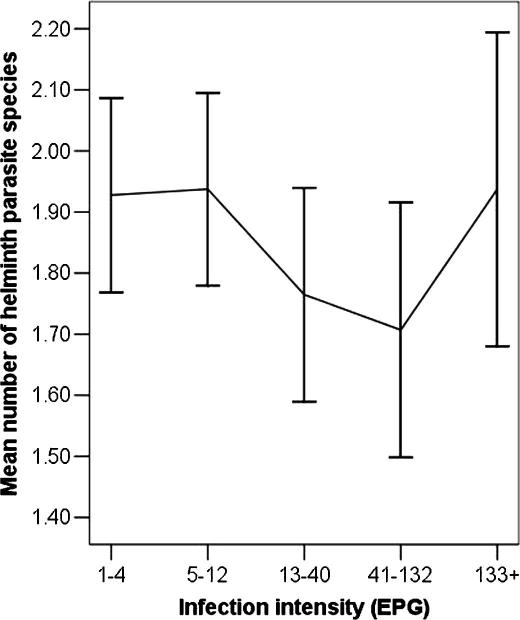
The number of helminth parasite species in individuals from the study population in the Poyang Lake, China, infected with differing infection intensities of *Schistosoma japonicum*.

Fig. 1 illustrates the mean number of helminth parasite species in individuals infected with *S. japonicum*. Individuals with low *S. japonicum* infection intensity were shown to have a higher mean number of parasite species. When a higher intensity of infection was experienced, the number of other helminth species decreased. The mean number of parasite species increased again in those with highest egg counts. These differences between the egg count groups were not significant (Kruskal–Wallis test; *P* = 0.25).

### 3.2 Parasite–parasite associations and covariates associated with infection status

There were no associations observed between *S. japonicum* and *A. lumbricoides* infections. However, there was a significant (*P* = 0.03) increase in risk of *T. trichiura* infection in an individual infected with *S. japonicum* (Odds Ratio = 1.28; 95% Confidence Interval (CI) = 1.03–1.60). Further, there was a strong association between *A. lumbricoides* infection and *T. trichiura* infection (*P* < 0.001; Odds Ratio = 4.43; 95% CI = 3.67–5.34).

Table 1 shows associations and risk factors for single, co- and multiple helminth infections. The youngest age group (5–14 years old) was at a significantly lower risk of infection with *S. japonicum* compared with older age groups. Gender, water contact and the number of past treatments were highly significant risk factors for *S. japonicum* infection. There was no age or gender association with *A. lumbricoides* infection; however, there was a significant association with water contact. Age and water contact were significant risk factors for *T. trichiura* infection. The highest *T. trichiura* prevalence (63.4%) was observed in the 5–14 years age group.

### 3.3 Variance components analysis of individual helminth infection

Four models were tested for each individual helminth infection: environmental, household, polygenic and general (household and polygenic). All models were compared with each other using the LRT. The favoured models for each infection, with corresponding parameter estimates, are provided in Table 2. A household model was favoured for *A. lumbricoides* indicating that 31.7% of risk of infection was attributable to domestic environment. No clustering of infection was observed at a familial level. *T. trichiura* infection was best described by the polygenic model; heritability was strong (29.9%) and significant (*P* < 0.05). Significant heritability was also estimated for *S. japonicum* infection (23.7%) as well as a strong household component (24.7%). The Kullback–Leibler *R*^2^ value estimated for all models was low, indicating that the covariates did not account for much of the variation in the models.

### 3.4 Variance components analysis of co- and multiple helminth infections

Given the strong association observed between *A. lumbricoides* and *T. trichiura* infection, a variance components analysis was undertaken to determine the factors underlying risk of having both infections concurrently (Table 3). Household effects were strongly significant (*P* < 0.05) and accounted for 32.7% of the risk of being infected with both parasites.

The association between *S. japonicum* and *T. trichiura* infection was also investigated. Again a household model was favoured with a strong effect size of 43.16% (*P* < 0.05; Table 3).

The variance components analysis estimated a heritability of 16.32% and a household effect of 8.8% for multiple helminthic infections. When both the household model and the polygenic model were compared to the general model (Table 3), both genetic and shared household effects remained significant in the presence of each other (*P* < 0.05).

## 4 Discussion

Helminth infections were common in this study population from the Poyang Lake region, China, and one third of the participants were found to harbour at least two parasite species concurrently. Hookworm prevalence was low and was not considered further in the analysis. As frequently observed with *S. japonicum* infections, males were more likely to be infected than females (Huang and Manderson, 1992, 2005; He et al., 1996). Water contact was also shown to be a major contributing factor for *S. japonicum* infection, reflecting the transmission characteristics of the parasite (Ross et al., 1998b; Li et al., 2000; Chen and Lin, 2004). Interestingly, water contact was a significant risk factor for individual infection, co-infection and multiple infections which would suggest water contamination as a common feature of the transmission of *S. japonicum*, *T. trichiura* and *A. lumbricoides*. A significant association was observed between *S. japonicum* and *T. trichiura* infection and between *A. lumbricoides* and *T. trichiura* infection. The latter association could be largely due to the similarity in transmission of these two nematode species which is closely related to poor hygiene behaviour and the lack of adequate sanitation (Booth et al., 1996; Tchuem Tchuenté et al., 2003; Raso et al., 2004a,b). The number of past treatments was also a significant indicator of current infection, suggesting those who had a high number of previous treatments were more prone to re-infection.

It is important to note that the results presented in Fig. 1 depict a trend as differences between the egg count categories were not statistically significant. However, a recent study in Brazil in individuals with concurrent *S. mansoni* and *A. lumbricoides* infection also indicated infection intensity for each parasite was lower than in those experiencing a single infection; a hypothesis was proposed suggesting that the similarity in the human immune response to both species may account for the decrease in parasite burden (Fleming et al., 2006). In contrast, however, our data (Fig. 1) suggest that those who appeared to be susceptible to extremely high levels of *S. japonicum* intensities also appeared to be highly susceptible to co- or multiple parasite infections, with many harbouring at least one other parasite. The association between very high infection intensity and polyparasitism has been described previously (Tchuem Tchuenté et al., 2003) and is suggestive that immunosuppression may be involved at this level of infection and this is an area that deserves further exploration. Further work to collect egg count data for STHs would also be of great interest to examine the correlations between different helminth infection intensities and to investigate the effect of poly-helminth infections on the associated clinical morbidity of individual infection.

Given the significant differences observed between helminth prevalences in the different villages, a village effect was incorporated into all the variance components analyses. The variance accounted for by other covariates in the model (Kullback–Leibler *R*^2^) was not shown to be high in any of the models and thus had little impact on the final parameter estimates. The variance components analysis for *A. lumbricoides* infection indicated household as a major contributing risk factor. In contrast to a previous study by Williams-Blangero et al. (1999), there did not appear to be any additive genetic effects involved. This is likely due to the binary phenotype used in this analysis, which decreased the power of the study to detect any aggregation at a familial level. Despite the loss of power due to the use of a binary phenotype (infection versus non-infection), significant heritability (29.9%) was still observed for *T. trichiura* infection although there was no aggregation at the household level. These findings are similar to those of Williams-Blangero et al. (2002a). Common domestic environment did appear to play a role in the risk of *S. japonicum* infection as well as a genetic involvement. Heritability remained high at 23.7% and was a significant risk factor; household was found to account for 24.7% of the risk of infection. These results are comparable with those of studies on *S. mansoni* (Bethony et al., 2001, 2002a).

The significant association identified between *A. lumbricoides* and *T. trichiura* infection was investigated by a variance components analysis in order to identify factors contributing to the risk of having a co-infection. The household model was found to be the most parsimonious and the household effect was surprisingly high (33.6%). This again may be explained, in part, by the commonality in the mode of transmission but, more notably, it could be a reflection of the socio-economic status of a particular household and the sanitation and hygiene conditions prevailing. Although data on household-specific risk factors were not collected (and merit further investigation) the results suggest that while mass chemotherapy is fundamental in the control of geo-helminths, an emphasis on the development of sanitation and clean water facilities, improved health and hygiene awareness, and more targeted treatment of those living in poor conditions is crucial to prevent transmission. Studies where sanitation and health awareness in a population have been improved and followed over time have shown marked decreases in prevalence that had been previously unattainable through chemotherapy alone (Esrey et al., 1991; Narain et al., 2000; Asaolu and Ofoezie, 2003), thus emphasising the value of using this combination method for control of STHs.

Variance components analysis of combined *S. japonicum/T. trichiura* infection also favoured a household model with an effect size of 43.2%. Despite the difference in transmission of these two parasites, water contact was a significant risk factor for both infections, again probably reflecting the lifestyle of the study communities. Although faecal matter is no longer used routinely as fertiliser in China, indiscriminate defecation is not uncommon (Bethony et al., 2002b; Davis et al., 2002). This suggests that control measures implemented for STHs in these villages would also reduce *S. japonicum* transmission. Further, a recent study of *S. mansoni* endemic communities in Brazil, has shown that adults in shared households of school children who were infected were also likely to infected (Massara et al., 2006). Targeting ‘infected households’ is thus an effective, rapid and practical method of identifying and treating helminth-infected adults in the community.

This investigation has shown that although the risk of infection with multiple helminth parasites was largely environmentally influenced, the genetic component was significant, accounting for 16.3% of the risk, and suggesting poly-helminth infections tends to aggregate in families. This would indicate that there may be common genetic components involved in susceptibility to multiple helminthic infections and that susceptibility to one parasite is not necessarily independent of susceptibility to another.

Overall, relatively few genetic studies have been conducted for susceptibility to helminth infections in human populations. Segregation and linkage studies have identified regions of interest for both *S. mansoni* (Marquet et al., 1996, 1999; Muller-Myhsok et al., 1997) and *A. lumbricoides* infections (Williams-Blangero et al., 2002b), although, to date, no such studies have been carried out on *T. trichiura* or *S. japonicum*.

We believe this study represents the first insight into the aggregation of multiple helminth infections in humans, suggesting there are common genetic factors involved in susceptibility to different parasitic helminth species, and it provides a foundation for future investigations of the genetics underlying this phenomenon. This will enable studies to be undertaken to identify genes or gene loci associated with susceptibility to helminth infection(s) and to identify possible gene–gene and gene–environment interactions involved in single and multiple infection events. As such, this genetic analysis may result in the possible identification of new drug targets and more targeted treatment of individuals susceptible to infection with parasitic helminths.

## Figures and Tables

**Table 1:** Association of single, co- and multiple helminthic infections, in the Poyang Lake study population using bi-variate logistic regression analysis

Parasite	*N*	Number (%) infected individuals	Association	Odds ratio	95% confidence interval	*P*-value^a^
*Schistosoma japonicum*			Age group			
	497	46 (9.8)	5–14	1		0.002
	246	45 (19.2)	15–24	2.20	1.41–3.43	
	369	60 (16.8)	25–34	1.87	1.24–2.82	
	538	95 (18.4)	35–44	2.09	1.43–3.05	
	365	61 (17.3)	45–54	1.93	1.28–2.91	
	435	62 (14.8)	55+	1.61	1.07–2.41	
			Sex			
	1032	123 (12.4)	Females	1		<0.001
	1428	247 (18)	Males	1.55	1.22–1.95	
	1670		Water contact	1.49	1.24–1.79	<0.001
	2452		Treatment number	1.03	1.01–1.05	<0.001
	2460	125 (5.1)	*A. lumbricoides*	1.17	0.93–1.48	0.19
	2460	193 (7.8)	*Trichuris trichiura*	1.28	1.03–1.60	0.03
						
*Ascaris lumbricoides*			Age group			
	497	167 (33.6)	5–14	1		0.08
	246	72 (29.3)	15–24	0.82	0.59–1.14	
	369	109 (29.5)	25–34	0.83	0.62–1.11	
	538	187 (34.8)	35–44	1.05	0.81–1.36	
	365	98 (26.8)	45–54	0.73	0.54–0.98	
	435	125 (28.7)	55+	0.80	0.60–1.05	
			Sex			
	1032	313 (30.3)	Females	1		0.57
	1428	448 (31.4)	Males	0.95	0.80–1.13	
	1670		Water contact	1.42	1.22–1.65	<0.001
	2460	550 (22.3)	*T. trichiura*	4.43	3.67–5.34	<0.001
						
*T. trichiura*			Age group			
	497	315 (63.4)	5–14	1		<0.001
	246	117 (47.6)	15–24	0.52	0.38–0.71	
	369	155 (42.0)	25–34	0.42	0.32–0.55	
	538	249 (46.3)	35–44	0.50	0.40–0.64	
	365	159 (43.6)	45–54	0.45	0.34–0.59	
	435	182 (41.8)	55+	0.42	0.32–0.54	
			Sex			
	1032	511 (49.5)	Females	1		0.2
	1428	669 (46.8)	Males	0.90	0.77–1.06	
	1670		Water contact	1.56	1.37–1.79	<0.001
						
*A. lumbricoides/T. trichiura*			Age group			
	497	139 (28)	5–14	1		0.07
	246	49 (19.9)	15–24	0.64	0.44–0.93	
	369	80 (21.7)	25–34	0.71	0.52–0.98	
	538	129 (24)	35–44	0.81	0.62–1.07	
	365	66 (18.1)	45–54	0.57	0.41–0.79	
	435	86 (19.8)	55+	0.64	0.47–0.86	
			Sex			
	1032	230 (22.3)	Females	1		0.93
	1428	320 (58.2)	Males	1.01	0.83–1.22	
	1670		Water contact	1.44	1.20–1.72	<0.001
						
*S. japonicum/T. trichiura*			Age group			
	497	37 (7.8)	5–14	1		0.38
	246	24 (10.1)	15–24	1.34	0.78–2.29	
	369	27 (7.5)	25–34	0.97	0.58–1.62	
	538	48 (9.2)	35–44	1.20	0.77–1.88	
	365	31 (8.7)	45–54	1.13	0.69–1.86	
	435	25 (5.9)	55+	0.74	0.44–1.25	
			Sex			
	1032	63 (6.3)	Females	1		<0.001
	1428	130 (9.4)	Males	1.53	1.12–2.10	
	1670		Water contact	2.20	1.61–3.02	<0.001
						
Multiple helminth species	2450		Age group			0.001^b^
	2460		Sex			<0.001^c^
	1670		Water contact			<0.001^d^

*N* = Total population with no missing data and infection phenotype.

a *P*-value based on likelihood ratio test.

b *P*-value based on Kruskal–Wallis test.

c *P*-value based on Student’s *t*-test.

d *P*-value based on linear regression.

**Table 2:** Summary of the favoured models of the variance components analysis for single, co- and multiple helminthic infections adjusted for significant covariates

Infection variable	Favoured model	Standardised parameter estimates			Kullback–Leibler *R*^2^⁎	Likelihood
		Genetic	Household	Environment		
*Ascaris lumbricoides*^a^	Household	(0)	0.317	0.683	0.078	−1196.863
*Trichuris trichiura*^b^	Polygenic	0.299	(0)	0.701	0.054	−819.762
*Schistosoma japonicum*^c^	General	0.237	0.247	0.516	0.023	−970.507
*S. japonicum/T. trichiura*^d^	Household	(0)	0.432	0.568	0.047	−381.677
*A. lumbricoides/T. trichiura*^e^	Household	(0)	0.336	0.664	0.013	−988.420
Multiple helminth species^f^	Polygenic	0.163	0.088	0.749	0.003	−253.742

a Covariates included in the model: water contact, *Trichuris* and administration village.

b Covariates included in the model: age, water contact, *A. lumbricoides*, *S. japonicum* and administration village.

c Covariates included in the model: age, sex, water contact, treatment number, *Trichuris* and administration village.

d Covariates included in the model: sex, water contact and administration village.

e Covariates included in the model: water contact and administration village.

f Covariates included in the model: age, sex, water contact and administration village.

⁎ The Kullback–Leibler *R*^2^ value denotes the proportion of variance due to the covariates in the models (excluding administration village).

**Table 3:** Results of variance components analysis for co- and multiple helminthic infections in the Poyang Lake study population

Hypothesis	*Ascaris lumbricoides*/*Trichuris trichiura* co-infection		*Schistosoma japonicum*/*Trichuris trichiura* co-infection		Multiple helminth infections	
	LRT	*P*-value	LRT	*P*-value	LRT	*P*-value
Household versus sporadic	49.116	<0.001	18.458	<0.001	23.545	<0.001
Genetic versus sporadic	27.925	<0.001	13.388	<0.001	24.146	<0.001
Household given genetic effects	21.19	<0.001	5.253	0.01	5.542	0.009
Genetic effects given shared household	0.038	0.42	0.183	0.33	6.143	0.007

LRT, likelihood ratio test.
